# Anhydrides-Cured Bimodal Rubber-Like Epoxy Asphalt Composites: From Thermosetting to Quasi-Thermosetting

**DOI:** 10.3390/polym8040104

**Published:** 2016-03-29

**Authors:** Yang Kang, Rui Jin, Qiang Wu, Liang Pu, Mingyu Song, Jixiang Cheng, Pengfei Yu

**Affiliations:** 1 College of Water Resources and Architectural Engineering, Northwest A&F University, Yangling, Shaanxi 712100, China; jinrui@nwsuaf.edu.cn (R.J.); wuqiang2011@nwsuaf.edu.cn (Q.W.); 18866800092@163.com (J.C.); 18382131697@163.com (P.Y.); 2 Department of Chemistry and Molecular Engineering, College of Science, Northwest A&F University, Yangling, Shaanxi 712100, China; lpu@nwsuaf.edu.cn (L.P.); mingyusong@nwsuaf.edu.cn (M.S.); 3 Jinan Urban Construction Group, Jinan, Shandong 250001, China

**Keywords:** linear viscoelastic (LVE), modified asphalt, epoxy asphalt, thermorheologically simple, bimodal networks

## Abstract

The present engineering practices show the potential that epoxy asphalt composites (EACs) would be a better choice to obtain long life for busy roads. To understand the service performance–related thermorheological properties of prepared bimodal anhydrides-cured rubber-like EACs (REACs), a direct tensile tester, dynamic shear rheometer and mathematical model were used. Tensile tests demonstrate that all the REACs reported here are more flexible than previously reported anhydrides-cured REACs at both 20 and 0 °C. The better flexibility is attributed to the change of bimodal networks, in which cross-linked short chains decreased and cross-linked long chains increased, relatively. Strain sweeps show that all the REACs have linear viscoelastic (LVE) properties when their strains are smaller than 1.0% from −35 to 120 °C. Temperature sweeps illustrate that the thermorheological properties of REACs evolve from thermosetting to quasi-thermosetting with asphalt content, and all the REACs retain solid state and show elastic properties in the experimental temperature range. A Cole–Cole plot and Black diagram indicate that all the REACs are thermorheologically simple materials, and the master curves were constructed and well-fitted by the Generalized Logistic Sigmoidal models. This research provides a facile approach to tune the thermorheological properties of the REACs, and the cheaper quasi-thermosetting REAC facilitates their advanced applications.

## 1. Introduction

Asphalt is a widely used paving material for its cohesiveness, driving comfort, lower cost and so on [1]. However, as a typical temperature-susceptible viscoelastic material, it flows at higher temperatures and becomes brittle at lower temperatures, especially with the increasing of heavier loads and traffic volumes as well as extreme climates [2,3,4]. Physical and chemical modifiers, e.g., styrene-butadiene-styrene (SBS), glycidyl methacrylate (GMA), *etc.*, have been employed to reduce the thermal susceptibility of asphalts [1,5,6,7]. Although these modifiers have improved the paving properties of base asphalt to some extent, they cannot meet the rigorous demands of long-span steel deck bridges because they are thermoplastic in essence, and thus, epoxy asphalt composites (EACs) have been developed [7]. EACs are entirely different from the traditional ones due to their thermosetting nature. Usually, they are two-component systems that result from the reaction of curing agents within asphalt (part A) and epoxy resin (part B) [8,9]. According to their curing agents, the EACs are categorized to be amine systems and acid systems [7,9,10,11]. Like the hydration reactions of cement-binding materials, once the two parts were mixed, the viscosity and modulus of the mixture would increase, and after a period of time (*i.e.*, pot life, usually greater than 45 min), it would be in the state of chemical gelation; lastly, a cross-linked binder formed. Usually, the resulting asphalt-filled epoxy cross-linked binder exhibits rubber-like mechanical properties in the neighborhood of room temperature. EACs have been widely used to pave steel deck bridges and other heavy loading traffic roads [12], and the present engineering practices show that EACs would be a better choice to obtain long life for busy roads [7,9].

However, the much higher prices of EACs compared to traditional modified asphalt binders thwart their applications, such as using them for pavement on highways, dams, high-speed railways and airports, *etc.* Also, the characterization method of EACs is borrowed from that of plastics, e.g., ASTM D 638-2010, Standard Test Method for Tensile Properties of Plastics. Obviously, to develop higher-performance/-cost EACs, it is important to understand their thermorheological properties, which are closely linked to the service performances of field applications. Thermorheological properties of asphalt binders are usually characterized by means of static experiments such as relaxation tests and creep tests, and dynamic experiments such as temperature sweeps or frequency sweeps under different conditions. These rheological data collected from dynamic experiments and static experiments in the linear viscoelastic (LVE) region can be mutually converted by means of the mathematical transformations [13,14,15]. Additionally, dynamic tests from a wide range are adopted widely because the procedures are time-saving. Moreover, rheological experimental data within the proper loading frequency range obtained from different temperatures can be shifted relative to the reduced frequencies for thermorheologically simple materials, *i.e.*, for which the time temperature superposition principle (TTSP) is suitable, so that the various curves can be aligned to form a single master curve [15], and then, the superposed master curve could be fitted by a mathematical function, which will be applied to the structure design of pavements.

In this paper, a direct tensile tester and dynamic shear rheometer were used to understand the mechanical and thermorheological properties of anhydrides-cured rubber-like EACs (REACs), which were prepared by the ideas of bimodal networks and quasi-thermosetting EACs [10,16,17]. LVE regions of the anhydrides-cured bimodal REACs were obtained by using strain sweeps at different temperatures. Temperature sweeps and frequency sweeps were conducted to understand the thermorheological properties of the REACs within LVE regions. A Cole–Cole plot and Black diagram were then depicted to verify whether REACs are thermorheologically simple materials or not, *i.e.*, whether TTSP can be used to produce master curves of the REACs or not. At last, a Generalized Logistic Sigmoidal model was presented for modeling the linear viscoelasticity of the REACs.

## 2. Materials and Methods

### 2.1. Materials

Maleic anhydride (Wuxi Resin, Wuxi, China), adipic acid (Yongzai Chemical, Zhejiang, China), methylhexahydrophthalic anhydride (Guanghui Hitech Chemical, Changzhou, China), Base Asphalt (90#, Shell, properties see Table 1, China), and epoxy resin (E-51, diglycidyl ether of bisphenol A, Wuxi Resin, Wuxi, China) were used as received.

### 2.2. Methods

#### 2.2.1. Preparation of the Bimodal Anhydrides-Cured REACs

**Figure 1:**
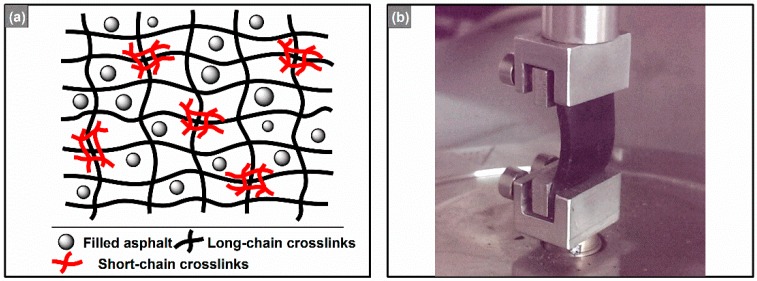
(**a**) Schematic diagram of bimodal networks; (**b**) Customized SRF5 solid torsion geometry.

The preparation method, which was inspired by the concept of bimodal networks (Figure 1a), was similar to the previous study [10]. In summary, reactions were carried out in a wide-mouthed glass flask fitted with a mechanical stirrer, a condenser (warm water refluxing, 75–85 °C), a gas inlet for N_2_ purge, and a thermocouple. First, maleic anhydride was added into the flask loaded with 150 °C asphalt (3.5:100, wt., total weight was 120.0, 150.0, 180.0, 210.0 and 240.0 g, respectively), and it was agitated for 4 h at 150 °C to obtain a maleated asphalt. Then, adipic acid and methylhexahydrophthalic anhydride (10:1, wt., total weight was 100.0 g) were poured into the flask subsequently. Finally, the mixture was stirred for about 0.5 h, and N_2_ was purged for an hour. The end product was designated as component A, and the epoxy resin E-51 as component B. Sample including 120.0, 150.0, 180.0, 210.0 and 240.0 g maleated asphalts per 100.0 g component A were labeled 120#, 150#, 180#, 210# and 240#, respectively.

#### 2.2.2. Characterization Procedures

To characterize the mechanical and thermorheological properties of the anhydrides-cured bimodal REACs, the well-weighed component A and B (3.5:1, wt.) were blended at 120 °C, and the mixture was sheared for one minute, then it was poured into a 2-mm-/3-mm-thick steel mold to cure for 4 h at 120 °C. Then, the cured bimodal REACs were prepared similarly to the references [10,16]. When cooled to the room temperature after 24 h, the material was cut into shapes that the following tests needed. To avoid the influences of heat history and stress history, each sample was discarded once used.

Direct tensile tests were carried out using a WDW-2000 Universal Tester (Changchun Kexin Test Machine, China) according to procedures of ASTM D638-2010 (type IV die) at the specific temperatures. Each sample was tested with six replicates.

Strain sweeps of the REACs were conducted by an MCR302 dynamic shear rheometer (Anton Parr Inc., Graz, Austria) with a customized SRF5 solid torsion geometry whose maximum shear stress is about 7.26 MPa, at an oscillation frequency of 10 rad/s, temperatures decreasing from 120 °C to −30 °C every 30 °C, and strains increasing from 0.001% to 10%. The sample dimensions are 10 mm × 10 mm × 2 mm. According to the SHRP-A-370 (Binder Characterization and Evaluation, Volume 4: Test Methods), when |G*| > 30 MPa, torsion geometry should be used. In fact, the customized SRF5 geometry was used because EACs are so hard (high moduli, especially at lower temperatures) that they are beyond the stress limits of the ordinary geometries, e.g., 8 mm plate-plate or 25 mm plate-plate. Furthermore, the solid REACs even have slipped from the scratched plates at negative temperatures. Hence, solid torsion geometry of SRF5 with higher stress limit and better clamping ability has been customized and calibrated by the engineers of Anton Paar (Figure 1b).

Temperature sweeps of the REACs were conducted by MCR302 with the customized SRF5 solid torsion geometry. The selected strain is 0.1%, oscillation frequency is 10 Hz, temperature range is from 120 °C to −30 °C, cooling rate is 1 °C/min, and the checking time continues for 10 min to achieve the thermal equilibrium state. According to Procedure A of ASTM D 618, which is referred by ASTM D 638, specimen should be at 23 ± 2 °C for 40 h to eliminate the thermal history and stress history. Based on the classical empirical Arrhenius equation of thermally-induced processes, we estimate that 10 min at 120 °C is enough to achieve the thermal equilibrium state. Especially, the dimensions of the specimens are 10 mm × 10 mm × 2 mm.

Frequency sweeps of the REACs were employed by MCR302 with the customized SRF5 solid torsion geometry. The selected strain is 0.1%, and the frequencies increase from 0.3 Hz to 300 Hz, temperatures decrease from 120 to 0 °C every 30 and −35 °C, and the temperature checking time is set to be 10 min, and the sample dimensions are 10 mm × 10 mm × 2 mm. Each rheological experiment mentioned above was reproduced with three replicates.

## 3. Results and Discussion

### 3.1. Direct Tensile Tests

**Figure 2:**
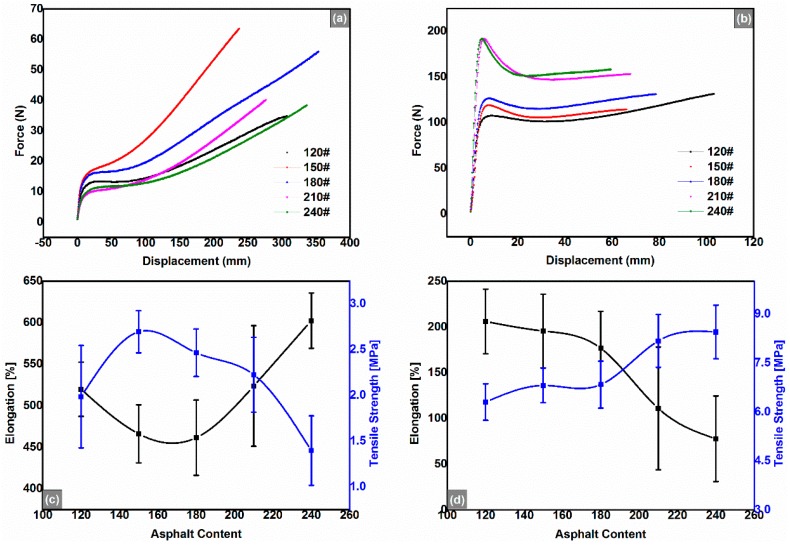
Force *vs.* displacement of the anhydrides-cured bimodal REACs at 20 °C (**a**) and at 0 °C (**b**); Tensile strength and rupture elongation *vs.* asphalt content at 20 °C (**c**) and 0 °C (**d**).

Asphalt binders are usually viscoelastic solids when temperatures are relatively lower, and with the temperature increasing, they become viscoelastic liquids, and at last they are Newtonian liquids. However, the REACs are elastic-plastic solids at lower temperatures, and with the temperature increasing, they become viscoelastic solids, and even at temperatures greater than 120 °C, they are still viscoelastic solids; thus, they are generally characterized by tensile tests that are borrowed from the characterization of plastics, for example ASTM D 638-2010: Standard Test Method for the Tensile Properties of Plastics. As shown in Figure 2a,b, the REACs exhibited typical non-Gaussian extension behaviors, *viz.* large upturns in the forces at high displacements; and previous studies have verified the bimodal networks of REACs (*i.e.*, “global-low-local-high” cross-linked networks, as shown in Figure 1a) by using Mooney-Rivlin plots, modified Mooney-Rivlin plots and microscope observations [10,16]. Moreover, all the REACs’ rupture elongation values were greater than 400% at 20 °C and greater than 50% at 0 °C (Figure 2c,d), which were more flexible than previously reported thermosetting anhydrides-cured bimodal REACs [16]. The better flexibility of the REACs reported here was attributed to the decrease of the weight ratio of maleic anhydride and base asphalt (*i.e.*, from 4.0:100 to 3.5:100); that is, with the decrease of the weight ratio, the cross-linked density of short-chain networks decreased. Meanwhile, the cross-linked density of long-chain networks relatively increased. With the asphalt content increasing, tensile strength values increased at first and then decreased when the asphalt content was higher than 150#; on the contrary, rupture elongation dropped at first and then was enhanced as shown in Figure 2c. However, at 0 °C, Figure 2d showed the different change tendencies of both rupture elongation and tensile strength as compared to Figure 2c. These forced high elastic properties, different from the elasticity when temperatures are higher than the glass transition temperature (*T*_g_), may be caused by the partially frozen cross-linked networks when the temperature is lower than its *T*_g_.

### 3.2. Linear Viscoelastic Regions

**Figure 3:**
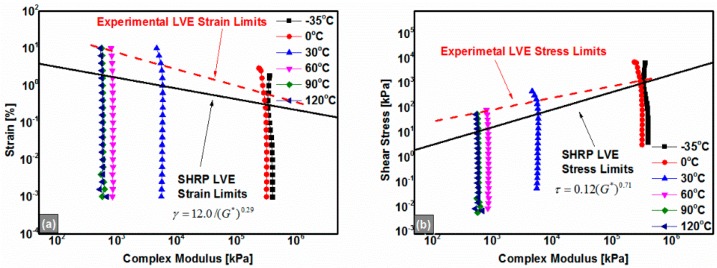
Strain sweep results of the anhydrides-cured bimodal REAC (take 180# for an example); (**a**) Strain *vs.* complex modulus (|G*|); (**b**) Shear stress *vs.* complex modulus (|G*|). All the anhydrides-cured bimodal REACs had the similar LVE limits.

To identify the LVE region in which the stress *vs.* strain behavior of the REAC is linear, strain sweep measurements were carried out. As shown in Figure 3, the 180# REAC retained its linear viscoelastic property when strains were smaller than 1.0% or the shear stresses were less than 5.0 × 10^4^ Pa for all temperatures. The experimental LVE results are consistent with the SHRP (Strategic Highway Research Program) stress and strain LVE limits as shown in Figure 3. The SHRP linear stress and strain criteria, which were made for penetration-grade bitumen materials, are functions of the complex modulus as defined by the following equations:

(1) $$\tau =0.12{\left(G^{*}\right)}^{0.71},$$

(2) $$\gamma =12.0/{\left(G^{*}\right)}^{0.29},$$

where τ is the shear stress kPa, and γ is the shear strain %, and G* is the complex modulus kPa. Obviously, the SHRP LVE limits are relatively conservative compared to the experimental results as shown in Figure 3. Thus, the strains were controlled to be 0.1% during all the thermorheological measurements to ensure they were in the LVE regions. Specially, all the anhydrides-cured bimodal REACs had the similar LVE limits.

### 3.3. Temperature Sweeps

**Figure 4:**
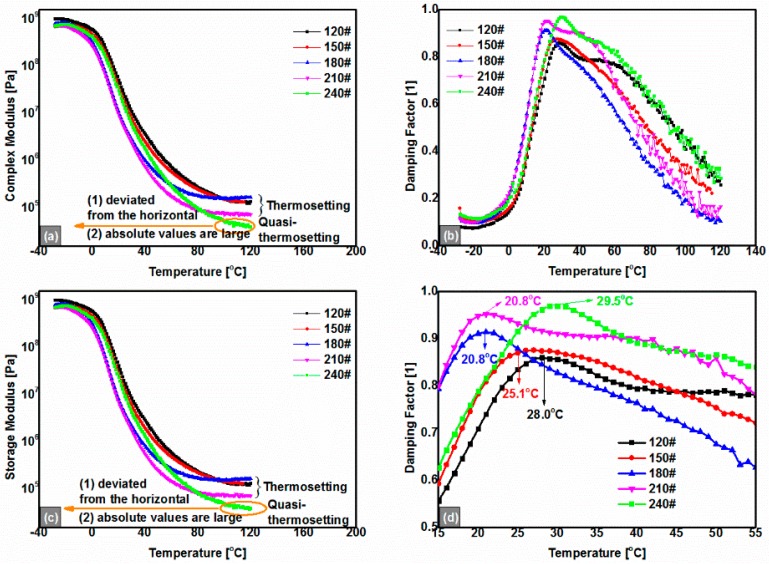
Temperature sweep results of the anhydrides-cured bimodal REACs with different asphalt content; (**a**) Complex moduli (|G*|) *vs.* temperature, REACs evolved from thermosetting to quasi-thermosetting; (**b**) Damping Factor *vs.* temperature; (**c**) Storage moduli (|*G*’|) *vs.* temperature, REACs evolved from thermosetting to quasi-thermosetting; (**d**) Damping Factor *vs.* temperature in a narrow temperature range.

As Figure 4a,c presented, in concert with the results of the tensile tests (Figure 2), all anhydrides-cured bimodal REACs exhibited elastic characteristics and retained solid state in the whole temperature range from −30 to 120 °C because the absolute values of the storage moduli (|*G*’|) were much greater than 1.0 × 10^4^ Pa and the damping factors (tan(δ) = |*G*’’|/|*G*’|) kept below 1.0 (*i.e.*, |*G*’| > |*G*’’|) simultaneously. The thermorheological properties ascribed to the bimodal networks resulted from the reactions of maleated asphalt, and organic anhydride with epoxy resin [10]. However, it is noticeable that, at temperatures approaching 120 °C, the complex shear moduli (|G*|) and storage moduli (|*G*’|) of 240# simultaneously decreased slightly to 2.0 × 10^4^ Pa, *i.e.*, their moduli did not keep constant at about 1.0 × 10^5^ Pa such as those of 120#, 150#, 180# and 210#. The findings are similar to that of the quasi-thermosetting polyetheramine-cured EACs [11]. Namely, there is also a limit of asphalt content to maintain the thermosetting nature for anhydrides-cured bimodal REACs. Surely, strictly speaking, thermosetting polymers are insoluble and infusible. However, owing to the thermoplastic nature of asphalt, it is impossible that thermosetting EACs are absolutely infusible and insoluble, for the asphalt filled within cross-linked epoxy networks would be melted and would flow from the cross-linked networks when temperatures are higher than 300 °C (Figure 1a); meanwhile, the service temperatures of asphalt concretes are far lower than 300 °C, and therefore, thermosetting EACs usually mean those whose |G*| keeps constant at much greater than 10 kPa when temperatures are approaching 120 °C. The |G*| of 10 kPa is arbitrarily selected for |G*| of SBS-modified asphalt binders at 120 °C, which are usually on the order of magnitude of 0.1 kPa; although the geometry and dimensions used to obtain these moduli of SBS-modified asphalt binder and EACs are different, the gap of two orders of magnitude of |G*| between the two asphalt binders is big enough to exhibit the thermosetting nature of EACs. Hence, quasi-thermosetting EACs mean those whose |G*| does not keep constant but gradually decreases to 10 kPa when temperatures are approaching 120 °C.

With the asphalt content increasing, the peak value of the damping factor enlarged as shown in Figure 4b; the corresponding *T*_g_, at which tan(δ) is maximum (Figure 4d), dropped at first and then increased. In our opinion, this is probably because unmaleated free asphalt was affected like a group of hard spheres filled into the chemical cross-linked bimodal networks, and then the plasticization effect enlarged the damping factor’s value, as shown in Figure 1a. However, peak widths of *T*_g_ enlarged with the asphalt content increasing, which implies that phase separation of the anhydrides-cured REACs occurred to some extent, especially in samples of 240#. Hence, thermorheological properties of 240# dropped to quasi-thermosetting from thermosetting compared with other lower-asphalt-content anhydrides-cured bimodal REACs.

### 3.4. Frequency Sweeps

**Figure 5:**
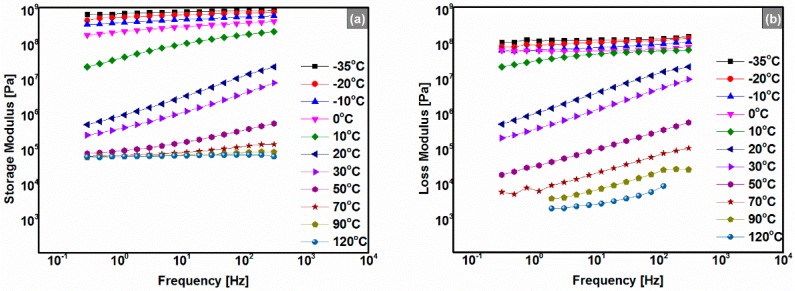
Frequency sweep results of the 180#; (**a**) storage modulus (|*G*’|) *vs.* frequency; (**b**) loss modulus (|*G*’’|) *vs.* frequency. All the anhydrides-cured bimodal REACs behaved similarly.

From the frequency sweep data shown in Figure 5 (take 180# REAC, for example), we found that |*G*’| and |*G*’’| of the anhydrides-cured bimodal REACs increased with the temperature decreasing and frequency increasing. When the temperatures are greater than 70 °C, values of |*G*’| keep constant; they also did so in the lower temperature range from −10 to −30 °C (Figure 5a). However, in the intermediate temperature range from 10 to 30 °C, |G’| and |G’’| decreased rapidly across three orders of magnitude with frequency, especially in the neighborhood of 10 to 20 °C (Figure 5c,d). These results indicated that there was a mechanical state transition (*i.e.*, *T*_g_) in this temperature zone, which is in agreement with the findings from the temperature sweep (Figure 4).

To obtain the linear viscoelasticity mathematical expressions of the anhydrides-cured bimodal REACs, the master curve should be produced first. Master curves can be produced by TTSP when the materials are thermorheologically simple and frequency sweeps are performed in the LVE region [18,19]. In other words, strictly speaking, master curve construction only makes sense if there are no macromolecular structural rearrangements within the concerned temperature range [20]. The Cole-Cole plot and Black diagram are two simple methods to justify whether the studied material is thermorheologically simple or not. That is, when the data of frequency sweeps overlap each other well to form a continuous smooth curve, the corresponding material is thermorheologically simple; on the contrary, if the two plots have bifurcations, it is thermorheological complex [19]. Also, the Black diagram and Cole–Cole plot are used to judge which mechanical model is suitable for simulating the paving material’s rheological behaviors [19]. Therefore, to evaluate the suitability of TTSP, the Cole–Cole plot and Black diagram were depicted, respectively.

### 3.5. Cole–Cole Plot and Black Diagram

**Figure 6:**
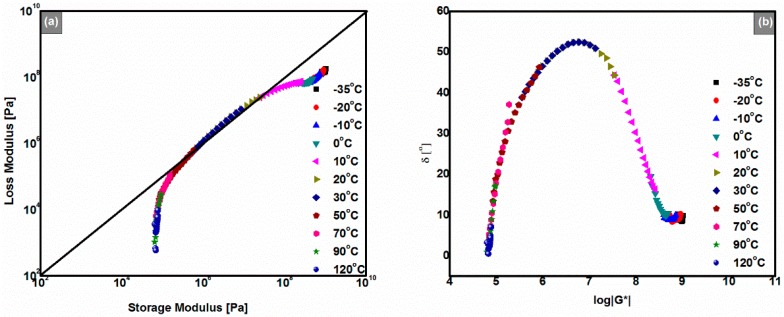
(**a**) Cole–Cole plot of the 180#; (**b**) Black diagram of the 180#.

As shown in Figure 6a, from the viewpoint of the overlap, the 180# REAC is thermorheologically simple in the experimental space. This result demonstrated that the master curve of the 180# REAC could be constructed by TTSP throughout the entire experimental temperature range. Furthermore, Figure 6a shows that, across the whole temperature range, |*G*’| were greater than |*G*’’|, which means 180# REAC had elastic properties from −35 to 120 °C. Additionally, all the REACs behaved similarly. These findings are in accordance with the results of the temperature sweeps (Figure 4).

The Black diagram, also named the van Gurp–Palmen plot or wicket plot, is another tool to judge whether a material is thermorheologically simple or not [19,20,21]. It is plotted by the phase angle (also named the loss angle) of the DSR data against the corresponding logarithmic values of the absolute complex modulus. As well as the Cole–Cole plot, when the TTSP holds, curves for different temperatures will overlap; otherwise, master curves cannot be produced by TTSP, which usually means materials are of thermorheological complexity [22]. Thus, we found the same conclusion more obviously as in the case of the Cole–Cole plot, as shown in Figure 6b. That is, in the whole experimental temperature range, TTSP validates the superposition of master curves of the anhydrides-cured bimodal REACs. Moreover, while phase angles of the 180# REAC stayed lower than 50° from −35 °C to 120 °C, |G*| were higher than 10^5^ Pa, as shown in Figure 6b.

In addition, as shown in Figure 5 and Figure 6, curves of |*G*’| and |*G*’’| at 10 °C and 20 °C spanned over about three orders of magnitude; at the same time, phase angle values of the Black diagram had a peak in the range of 10 to 20 °C (Figure 6b). According to the theory of glass transition, we concluded that the glass transition temperature of the 180# REAC was in the range of 10 to 20 °C (*i.e.*, 20.8 °C as shown in Figure 4d). Also, all the anhydrides-cured bimodal REACs behaved similarly. The glass transition temperature found here is equivalent to that from the former temperature sweeps (Figure 4).

### 3.6. Master Curves

**Figure 7:**
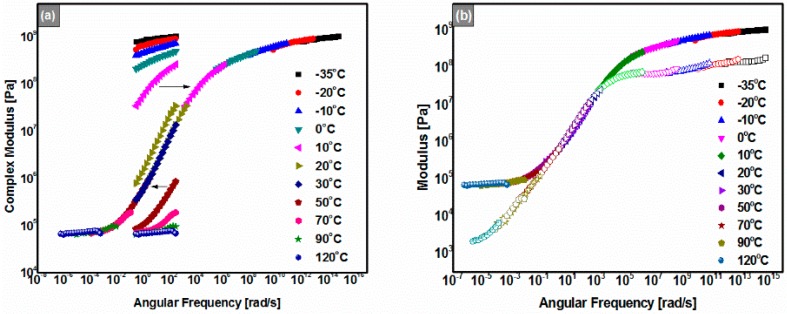
Master curves of the 180# (*T*_ref_ = 30 °C); (**a**) Complex modulus (|G*|) *vs.* frequency; (**b**) Storage modulus (|*G*’|) (solid interior symbols) and loss modulus (|*G*”|) (open interior symbols) *vs.* frequency.

Figure 7 shows the master curve of the 180# REAC superposed on the frequency sweep results by TTSP. The 180# REAC exhibited elastic properties in the whole range of frequency at the reference temperature of 30 °C for |*G*’| ≥ |*G*’’| throughout the entire reduced frequency range. When angular frequencies approached 0, |*G*’| and |G*| retained values higher than 0.1 MPa; this implies that 180# REAC has superior low-frequency or long-time performance. Especially, the whole plot looks like the mirror of its temperature sweep result as shown in Figure 4a,c, which could be seen as an embodiment of TTSP. Furthermore, Figure 7 shows that there were two asymptotes of the modulus under the lowest and highest frequencies, respectively. It is worthy of notice that the results of the low-frequencies region (long-time zone) are quite different from those of the traditional thermoplastic polymer-modified asphalt binders including SBS-modified asphalt binders [4]. The differences are believed to be attributed to the structure difference between chemical cross-linked bimodal network structures and colloidal structures.

### 3.7. Generalized Logistic Sigmoidal Model

Many mathematical models have been built to properly describe types of master curves of asphalt binders and their concretes. These models can be classified into two groups: empirical algebraic equations and mechanical element models (or analogical models). There is no essential distinction among these empirical models. They were created just for the different shapes of rheological master curves; in other words, they are selected by the nature of the studied asphalt binders or asphalt concretes, *i.e.*, solids or liquids. The generalized Maxwell and generalized Kelvin, Huet, Huet-Sayegh and 2S2P1D are the main mechanical element models [23,24]. The most popular empirical algebraic models are the Christensen and Anderson (CA) model, Christensen, Anderson and Marasteanu (CAM) model, modified CAM model [25] and the Logistic Sigmoidal model [26].

**Figure 8:**
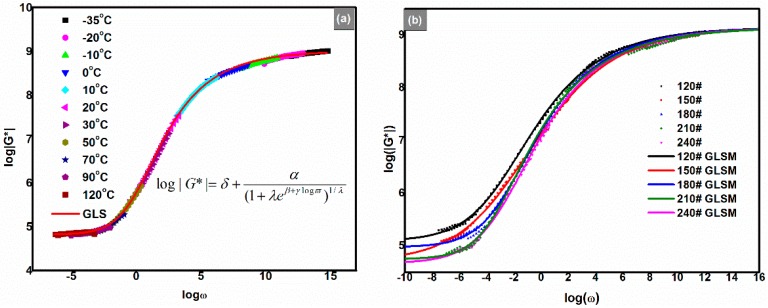
(**a**) Complex modulus (|G*|) master curve of the 180# fitted by the GLS model in the temperature range from −35 to 120 °C; (**b**) Experimental data with the GLS model simulated function curves (*T*_ref_ = 30 °C).

Obviously, the shape of the |G*| master curve is asymmetric sigmoidal, which differs from those of base asphalt binders and traditional polymer-modified asphalt binders. Therefore, functions of the sigmoidal type are preferred to be selected. Moreover, a standard sigmoidal logistic model has been adopted as part of the AASHTO MEPDG (Mechanistic Empirical Pavement Design Guide) [25,26]. To describe the asymmetric sigmoidal master curves of filled polymer mastics materials, a generalized logistic sigmoidal (GLS) model was introduced by Rowe *et al.* [27]. This equation is also applicable to asphalt binders and the model can be written as the following:

(3) $$\log\left|G^{*}\right|=\delta +\frac{\alpha }{{\left(1+\lambda e^{\left(\beta +\gamma \log\varpi \right)}\right)}^{1\backslash \lambda }},$$

where logϖ is the logarithmic reduced angular frequency, δ is the lower absolute complex modulus asymptote, α is the difference between the values of the upper and lower absolute complex modulus asymptote, β and γ define the shape between the asymptotes and the location of the inflection point (inflection point obtained from logϖ = −β/γ, when λ = 1, the point satisfied with β + γlogϖ = 0), *λ* is the non-symmetrical shape coefficient of the master curve (based on logϖ = 0). When *λ* is reduced to 1, Equation (3) is reduced to the standard logistic sigmoidal model [26]. Figure 8 shows that the GLS model fits the rheological data very well, and the fitted parameters are listed in Table 2. The parameter of the highest logistic value of the |G*| asymptote was arbitrarily set to be a constant at 9.15, according to the temperature sweep results shown in Figure 4a.

## 4. Conclusions

Tensile tests indicated that all the bimodal REACs exhibit similar high elastic or forced high elastic properties in that all their rupture elongation values are greater than 400% at 20 °C or greater than 50% at 0 °C, respectively. In comparison with previously reported anhydrides-cured REACs, the better flexibility of the REACs reported here is attributed to the decrease of cross-linked short-chains and the relative increase of cross-linked long-chains, which originates from the weight ratio drop of maleic anhydride and base asphalt. Temperature sweeps illustrated that thermorheological properties of the REACs evolve from thermosetting to quasi-thermosetting because their absolute complex moduli and absolute storage moduli do not keep constant but drop, to some extent, with an increase of asphalt content, and all the REACs retain solid state and exhibit elastic properties in the overall experimental temperature range. Strain sweep results showed that all the REACs have LVE properties when the strains are smaller than 1.0% from −35 to 120 °C. The Cole–Cole plot and Black diagram of all the REACs indicated that, throughout the entire experimental temperature range, they are thermorheologically simple materials, and their master curves can be superposed by TTSP. All these properties are ascribed to their unique chemical cross-linked bimodal epoxy-anhydrides networks, which are different from the traditional colloidal structures of thermoplastic modified asphalt binders. Specially, in concert with the temperature sweep results, the master curve shapes of the REACs are not similar to those of “liquid” traditional thermoplastic polymer-modified asphalt binders but are similar to those of “solid” asphalt concretes. Therefore, they can be fitted only by mathematic models for “solids”. For the convenience of engineering applications, the superposed master curves of all the REACs were perfectly fitted by the phenomenological Generalized Logistic Sigmoidal models.

## Figures and Tables

**Table 1:** Specification of base asphalt.

Properties of asphalt		Method
Oil Source	Saudi Arabia	
Density@ 15 °C/g·cm^−3^	1.013	JTJ 052 T0603-2011
Penetration@ 25 °C/0.1 mm	88.0	JTJ 052 T0604-2011
Soft Point (°C)	46.5	JTJ 052 T0606-2011
Viscosity@60 °C/Pa·s	225.0	JTJ 052 T0625-2011
Ductility@ 15 °C/cm	>100	JTJ 052 T0624-2011
Flash point (°C)	308	JTJ 052 T0611-2011
Fraass breaking point (°C)	−17.8	JTJ 052 T0613-1993
SARA		JTJ 052 T0618-1993
Saturate (%)	23.8	
Aromatic (%)	37.2	
Resin (%)	22.1	
Asphaltene (%)	16.9	

**Table 2:** Parameters fitted by the generalized logistic sigmoidal GLS model.

No.	δ	α	β	γ	λ	*R*^2^
120#	5.096	4.054	0.846	0.543	2.097	0.9997
150#	4.714	4.436	0.394	0.394	1.480	0.9993
180#	4.981	4.169	0.854	0.671	2.610	0.9995
210#	4.753	4.397	1.455	0.776	3.301	0.9991
240#	4.678	4.472	0.829	0.618	2.454	0.9988
